# Real-Time Facial Affective Computing on Mobile Devices

**DOI:** 10.3390/s20030870

**Published:** 2020-02-06

**Authors:** Yuanyuan Guo, Yifan Xia, Jing Wang, Hui Yu, Rung-Ching Chen

**Affiliations:** 1 Institute of Smart Healthcare Systems, Qingdao Academy of Intelligent Industries, Qingdao 266109, China; yuanyuan.guo@qaii.ac.cn; 2 School of Creative Technologies, University of Portsmouth, Portsmouth PO1 2DJ, UK; yifan.xia@myport.ac.uk; 3 Institute of Automation, Chinese Academy of Sciences, Beijing 100190, China; 4 Department of Information Management, Chaoyang University of Technology, Taichung 41349, Taiwan; crching@cyut.edu.tw

**Keywords:** facial affective computing, convolutional neural networks, deep learning, mobile development

## Abstract

Convolutional Neural Networks (CNNs) have become one of the state-of-the-art methods for various computer vision and pattern recognition tasks including facial affective computing. Although impressive results have been obtained in facial affective computing using CNNs, the computational complexity of CNNs has also increased significantly. This means high performance hardware is typically indispensable. Most existing CNNs are thus not generalizable enough for mobile devices, where the storage, memory and computational power are limited. In this paper, we focus on the design and implementation of CNNs on mobile devices for real-time facial affective computing tasks. We propose a light-weight CNN architecture which well balances the performance and computational complexity. The experimental results show that the proposed architecture achieves high performance while retaining the low computational complexity compared with state-of-the-art methods. We demonstrate the feasibility of a CNN architecture in terms of speed, memory and storage consumption for mobile devices by implementing a real-time facial affective computing application on an actual mobile device.

## 1. Introduction

Facial affect plays a crucial role in our daily lives such as psychological analysis, medical diagnosis, education, decision-making, customer marketing, and advertising. Driven by the vast application demands, facial affective computing has become an active research field and has attracted a lot of research attention from various research areas such as human-computer interaction, computer vision and artificial intelligence. In particular, facial affective computing is one of the most important components of human-computer interaction, because it provides a new dimension to human-machine interactions. For instance, if robots can analyze human facial affect, they can have appropriate responses and behaviors according to the analysis results.

In the field of psychology, affect is a term for the external exhibition of internal emotions and feelings. The aim of facial affective computing is to develop algorithms or systems to interpret and estimate human affects from human facial images or videos [1]. Specifically, facial affect is usually described based on two types of models: one is the categorical model, namely facial expressions, such as the six basic facial expressions (Happiness, Sadness, Fear, Anger, Surprise and Disgust) defined by Ekman et al. [2]; another is the dimensional model which uses valence and arousal to represent the facial expression intensity on a continuous scale. Valence distinguishes the degree of positive or negative of a facial expression, and arousal indicates the degree of intriguing/agitating or calming/soothing of an event [3].

Most research about facial affective computing has mainly focused on constrained laboratory environments instead of real-world scenarios and is not suitable for large-scale practical applications. Due to recent advances in deep learning technologies and the advent of data-bases which are large-scale and in-the-wild, convolutional neural networks (CNNs) have obtained remarkable performance in facial affective computing and outperformed many conventional methods with a large margin. However, one of the drawback of the CNNs is that the computational complexity increases significantly as the performance improves. Therefore, CNNs are usually performed on high-performance devices. But for ordinary users, these devices are expensive and not portable enough. Moreover, many users usually have no opportunity to access these devices.

Recently, mobile devices with embedded cameras have become inseparable parts of people’s lives, which play an important role in many personal and business applications such as video chat and social networks. Moreover, a wide variety of emerging mobile applications have been actively studied in various areas such as human-computer interaction, education and entertainment. The popularity and portability of mobile devices with high-quality cameras motivate us to develop real-time facial affecting computing on mobile devices using CNNs for ordinary users. However, conventional CNNs are not easy to implement and generalizable enough for real-time applications on mobile devices where the storage, memory and computational power are relatively limited. Therefore, it is of central importance to design a novel CNN architecture for facial affective computing on mobile devices.

The aim of this paper is to investigate the possibility of CNNs embedded in mobile devices for real-time facial affective computing under real-world scenarios. The main challenge in this task is the processing of the users’ facial images on the device itself, without uploading the images to the external cloud server for processing. There are two advantages to do this: one is that it is beneficial for the security of user information and privacy and the other is that compared with uploading to the cloud server, the processing speed is faster on the mobile devices since no data is uploaded through the network. Major contributions of our work are as follows: (1) To overcome the limited processing power of mobile devices, we propose a light-weight but effective CNN architecture for real-time facial affective computing using both categorical model and dimensional model on mobile devices, which well balances the performance and computational complexity; (2) We have explored facial affective computing using both categorical model (e.g. happy, neutral) and dimensional model (valence and arousal) on mobile devices. (3) The proposed network has been implemented on mobile devices with an embedded mobile camera and requires only a low consumption of memory and storage. The implemented application can analyze users’ facial affect in real-time, which can be a good functional component for other emerging mobile applications.

The structure of this paper is as follows. In Section 2, we introduce the related work about facial affective computing. Section 3 describes the proposed light-weight CNN architecture in detail. Section 4 provides the experimental results of the proposed method on the challenging database. We show the performance of implemented facial affective computing mobile application in Section 5 and Section 6 is the general discussion. Finally, we conclude this paper and give future works in Section 7.

## 2. Related Work

Many studies in facial affective computing have been published over the past few years [4,5,6] since facial affect plays an important role in human-computer interaction [7,8,9]. For example, facial affect computing is a functional supplement for intelligent surveillance [10] when combining with multiple moving targets tracking technologies [11] to detect the facial affect of the crowd in video surveillance to avoid potential dangers and disasters. Previous works mainly focused on the categorical model based on six basic facial expressions (Happiness, Sadness, Fear, Anger, Surprise and Disgust) defined by Ekman et al. [2]. The extracted features are applied to the classifier such as support vector machines (SVM) [12,13], AdaBoost [14] and hidden Markov models (HMMs) [15] to achieve facial expression recognition. Traditional methods mainly depend on hand-crafted features based on facial information such as geometry, appearance or texture information. Therefore, according to the different facial information, the features for facial expression recognition can be roughly divided into two categories: geometric features and appearance features.

Geometric features describe the locations and shapes of facial components extracted from facial images such as measurements among coordinates of landmarks on the face. Active Appearance Model (AAM) [16] and the Active Shape Model (ASM) [17] are used in most geometric feature-based methods for detecting facial components, for instance, Choi et al. [18] proposed a real-time facial expression recognition method used AAM with second order minimization and a neural network. Appearance features use the texture information of the face, including a histogram of oriented gradients (HoG) [19,20], local binary pattern (LBP) [12,21], scale invariant feature transform (SIFT) [22,23], and Gabor features [24,25]. However, these hand-crafted features can be implemented on facial expression recognition under laboratory-controlled environment successfully but are not generalizable enough for the variation from the wild environment such as lighting, camera view, head pose and ethnicity.

In recent years, deep learning, especially convolutional neural networks (CNNs), has achieved impressive performance for various computer vision and pattern recognition tasks [26,27,28,29]. There are also enormous methods using CNNs in facial expression recognition in recent years [30,31,32]. In addition, there also have been some researches on the dimensional model since the development of deep learning [33,34]. Within the dimensional model, a particular facial affect can be mapped on an arousal/valence value, which describes the range of arousal (high to low) and valence (pleasure to displeasure). In these studies, deep learning-based architectures are especially efficient for facial affective computing and can achieve high performance. The main reason for the success of CNNs is that CNNs are able to overcome the limitations of the handcrafted features in the wild settings by directly extracting highly discriminative features of the raw data.

However, existing CNNs are not generalizable enough for mobile devices, since they were not originally designed for mobile devices and didn’t consider the storage, memory and computational power of mobile devices as important attributes in the architecture design. Real-time computing on mobile platforms is challenging because of hardware performance constraints. To the best of our knowledge, for mobile affective computing, existing approaches are mainly limited to use one categorical model for facial expression recognition [35]. FaceReader [36] is a successful and commercially available software program that can automatically analyze facial expressions using the categorical model. But for the dimensional model, FaceReader can only perform the valence prediction on mobile platforms and the arousal prediction is still unavailable currently. Therefore, real-time facial affective computing using both categorical model (e.g. happy and neutral) and dimensional model (valence and arousal) are barely used for mobile platforms. To overcome these limitations, we design a light-weight CNN architecture for facial affective computing on mobile devices. The proposed architecture well balances performance and computational complexity. We have implemented the proposed network architecture on actual mobile devices to demonstrate its feasible and high efficiency. The implemented mobile application can analyze users’ facial affect and output both users’ facial expression category and values of valence and arousal in real-time.

## 3. Facial Affective Computing

**Figure 1:**
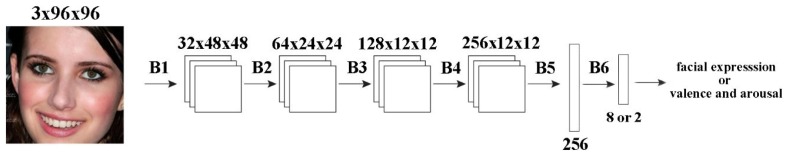
The architecture of our light-weight deep model for facial affective computing (the facial image is from the AffectNet [33]). The network consists of four convolutional blocks, where the raw image pixel is treated as the input.

We propose a light-weight CNN architecture for facial affective computing. The network architecture is shown in Figure 1. In this section, we firstly provide instruction of the proposed network structure and then discuss the loss functions used in the training stage.

### 3.1. Network Architecture

This work mainly focuses on designing a light-weight CNN for facial affective computing on mobile devices, which uses facial images as input and outputs the categories of facial expression and values of valance and arousal. We start with VGG network [37] for facial affecive computing to create our network. Unlike other conventional CNNs, VGG network consists of several convolutional blocks, each of which contains several convolutional layers stacked on top of each other. These convolutional layers have a very small kernel size of 3 × 3 with the stride of 1. And there is a Maxpooling layer which often has a pool size of 2 × 2 with a stride of 2 at the end of each convolutional block. The convolutional layers in the same block often have the same number of filters.

The proposed light-weight CNN is inspired by the VGG network [37], which can be regarded as a simplified VGG network with the attributes of few parameters and low computation complexity but high performance. These attributes of the proposed network architecture meet the requirements of mobile development. The main design principle of the proposed network architecture is to reduce the computation complexity and parameters of the original VGG network. In fact, we strongly reduce the number of parameters in all layers compared with the original VGG network. The architecture of the proposed network is shown in Figure 1 and Table 1. The proposed network architecture contains four convolutional blocks. For each convolutional block except the first and last one, there are three convolutional layers and one Maxpooling layer. The first convolutional block has two convolutional layers and one Maxpooling layer. And the last convolutional block only contains three convolutional layers. Batch normalization [38] is also used after each convolutional layer to improve the training process. The numbers of filters of each convolutional layer of four convolutional blocks are set to 32, 64, 128 and 256 respectively.

**Figure 2:**
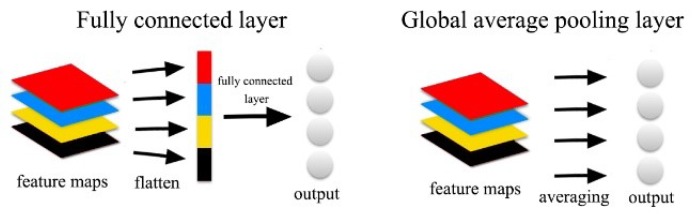
Difference between fully connected layer and global average pooling layer.

After four convolutional blocks, we use a Global Average Pooling (GAP) layer instead of fully connected layers to extract 256-dimensional feature vectors. The purpose of using GAP is to reduce parameters and computation complexity. GAP was proposed by Lin et al. [39]. In [39], which was used to takes the average of each feature map replacing the traditional fully connected layers on the CNN. As shown in Figure 2, the main difference between those two layers is that the output of a flatten layer has to be fed into a fully connected layer to get features we need, but the output of the global average pooling layer has already the desired dimension. The fully connected layer can add a large number of parameters and then increase the complexity of the model and the degree of overfitting. Therefore, the main advantage of GAP over the fully connected layers is that there is no need for parameter optimization in the global average pooling, which greatly reduces parameters and computation complexity.

Finally, the facial expression recognition and prediction of valence and arousal in this paper are regarded as a classification and regression task respectively. And the proposed light-weight CNN architecture is used for both classification and regression tasks. Therefore, after GAP, the output layer of the network contains 8 nodes with softmax activation or 2 nodes with linear activation depending on the tasks of classification or regression.

### 3.2. Loss Function

In this work, we use both the categorical model (e.g. happy, neutral) and dimensional model (valence and arousal) to describe the facial affect. For facial expression recognition, we regard it as a multi-class classification problem. And we formulate the task of valence and arousal estimation as a regression problem. Therefore, the loss function for the task of facial affective computing can be represented as the formula:

(1) $$L_{Affect}=\left\{{loss}_{c},{loss}_{r}\right\}$$

where ${loss}_{c}$ and ${loss}_{r}$ represent the loss function for the task of classification and regression respectively, and next we will introduce them in detail.

**Facial expression recognition:** For multi-class classification problems, Categorical Cross Entropy (CCE) is a commonly used loss function as fol-lows:

(2) $${loss}_{CCE}=-\sum_{i=1}^{N}g_{i}\log\left(p_{i}\right)$$

where *N* is the number of images in the training data; $p_{i}$ and $g_{i}$ represent the prediction and ground truth. However, CCE can lead to over-fitting problem and make the model become too confident about its predictions. Therefore, we modify the CCE loss function using the Label Smoothing Regularization [40], the variant of CCE loss function is thus given by:

(3) $${loss}_{c}=-\left(1-\varepsilon \right){loss}_{CCE}-\varepsilon H_{u,p}$$

where ε is a hyper parameter, it is set to 0.1; $H_{u,p}$ represents the dissimilarity between the predicted distribution p and its uniform distribution, defined as:

(4) $$H_{u,p}=\sum_{i=1}^{N}\frac{1}{C}p_{i}\log\left(p_{i}\right)$$

where *C* is the number of categories (*C* = 8 in AffectNet database [33]).

**Valence and arousal estimation:** Mean Squared Error (MSE) loss function is usually used for regression problem which shown as follows:

(5) $${loss}_{MSE}=\frac{1}{N}\sum_{i=1}^{N}{\left(p_{i}-g_{i}\right)}^{2}$$

where *N* is the number of images in the training data; $p_{i}$ and $g_{i}$ represent the prediction and ground truth of valence and arousal of each facial image. However, since range of the value of valence and arousal are from −1 to 1, it can lead to inaccurate estimation if we only use MSE, for instance, the prediction values of valence are −0.2 and 0.6 respectively, they have the same RMSE if the ground truth is 0.2, but prediction of 0.6 is better than prediction of −0.2 since prediction of 0.6 expresses a positive emotion similar to the ground truth. Therefore, we modify the MSE loss function giving more punishment to the samples with different signs, the variant of MSE loss function which shown as follows:

(6) $$f=\left\{\begin{array}{cc} {loss}_{MSE}, & S(p_{i},g_{i})=0\\ {loss}_{MSE}+\alpha \frac{1}{N}\sum_{i=1}^{N}sign(S\left(p_{i},g_{i}\right)), & S(p_{i},g_{i})\neq 0 \end{array}\right.$$

where α is hyper parameter, it is set to 0.1; $S(p_{i},g_{i})$ is defined as:

(7) $$S\left(p_{i},g_{i}\right)={\left(sign\left(p_{i}\right)-sign\left(g_{i}\right)\right)}^{2}$$

where sign(·) is the sign function.

## 4. Experiment

### 4.1. Experimental Setup

**Figure 3:**
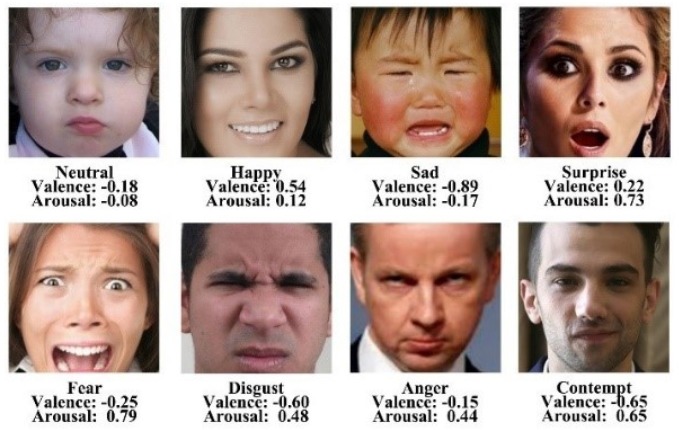
Examples of the eight categories of facial expressions (Neutral, Happy, Sad, Surprise, Disgust, Fear, Anger and Contempt) and their values of valence and arousal from the AffectNet [33].

One of the key issues for implementing facial affective computing using CNNs is the choice of database. Most existing databases for facial affective computing in the wild are so small and only contain annotated image data of the categorical model (facial expressions). There are few databases for facial affective computing providing annotated image data of the dimensional model (valence and arousal). For the proposed light-weight CNN training, we use by far the largest database in the field of facial affective computing, AffectNet [33], which provides annotated the categorical model and dimensional model image data. This database includes more than 1M facial images, which its creators collected them from three major search engines by using 1250 related keywords in six different languages. About 420,299 manually annotated facial images have labels of categories of facial expressions and the values of valence and arousal. Figure 3 shows examples of annotated facial images from the AffectNet database.

For facial expression recognition, we regard it as a multi-class classification problem. The numbers of each category of facial expressions for training is listed in Table 2. During the process of training, we use eight categories of facial expressions for training including Neutral, Happy, Sad, Surprise, Fear, Disgust, Anger and Contempt and invalid facial expressions (none, uncertain and no-face) in Affectnet database are discarded. The total number of facial images for facial expression classification in the training set is 287,651. We formulate the task of valence and arousal estimation as a regression problem in which the network learns to predict the values of valence and arousal from a face image. In the AffectNet database, the facial images are manually annotated for the values of valence and arousal from −1 to 1. The facial images labeled with −2 are invalid data in the database which we also discard. The total number of training data is 320,739.

During the stage of preprocessing, we only use manually annotated images that are divided by its creators into training and validation sets and contains about 420,299 images. The images of the AffectNet database are cropped to the size of a face bounding box provided by the database. And then we resize all the cropped face images to be an equal size of 96 × 96 px. In our experiment, we train two separate light-weight CNNs for facial expression recognition and valence and arousal estimation. The proposed light-weight CNN is trained on a desktop PC with a specification of Intel (Santa Clara, CA, USA) Core i7, 4.20 GHz processor, 16 GB of RAM memory and 8 GB NVIDIA (Santa Clara, CA, USA) GeForce GTX 1080 GPU. All tasks are trained end-to-end using TensorFlow. For all tasks, we use the stochastic gradient descent (SGD) method to optimize the model and set momentum as 0.9. The initial learning rate is set as 0.01 and divided by 10 after 15 epochs. We stop training in 30 epochs. During the training stage, we set the batch size as 128.

### 4.2. Evaluation Metrics

We evaluate the proposed light-weight CNN on the AffectNet validation set since currently its test set has not been released. The total number of facial images for facial expression classification and valence and arousal estimation in the validation set are 4000 and 4500 respectively. For facial expression classification, we use classification accuracy as the main evaluation metric since it is well-defined widely used metrics for evaluation of the classification task. And for the valence and arousal estimation, we use and calculate 4 different evaluation metrics for evaluation of valence and arousal estimation task, since it outputs the values of valence and arousal in a continuous domain. In the following, we briefly review these metrics.

One of the the most common evaluation metric in a continuous domain is Root Mean Square Error (RMSE) which is defined as:

(8) $$RMSE=\sqrt{\frac{1}{n}\sum_{i=1}^{n}{\left(p_{i}-g_{i}\right)}^{2}}$$

where *n* is the number of images in the evaluation set; $p_{i}$ and $g_{i}$ are the prediction and ground truth of *i* th image.

Pearson’s Correlation Coefficient (CC) [41] is another evaluation metric which can consider the covariance of prediction and ground-truth compared with RMSE:

(9) $$CC=\frac{COV\{p,g\}}{\sigma_{p}\sigma_{g}}$$

where COV is covariance function; $\sigma_{p}$ and $\sigma_{g}$ are the standard deviation of each time series (e.g., prediction and ground-truth).

Based on CC, Concordance Correlation Coefficient (CCC) [42] computes the square difference between the means of two compared time series:

(10) $$CC=\frac{2\rho \sigma_{p}\sigma_{g}}{{\sigma_{p}}^{2}+{\sigma_{g}}^{2}+{\left(\mu_{p}-\mu_{g}\right)}^{2}}$$

where ρ is CC; $\sigma_{p}^{2}$ and $\sigma_{g}^{2}$ are the variance of each time series. Unlike CC, the predictions that are well correlated with the ground-truth but shifted in value are penalized in proportion to the deviation in the CCC.

Sign Agreement (SAGR) is a very important evaluation metric proposed in [41] to evaluate the performance of a valence and arousal estimation with respect to the sign agreement. Therefore, SAGR is defined as:

(11) $$SAGR=\frac{1}{n}\sum_{i=1}^{n}\delta (sign\left(p_{i}\right),sign\left(g_{i}\right))$$

where δ is the Kronecker delta function, defined as:

(12) $$\delta \left(a,b\right)=\left\{\begin{array}{cc} 1, & a=b\\ 0, & a\neq b \end{array}\right.$$

### 4.3. Experimental Results

**Figure 4:**
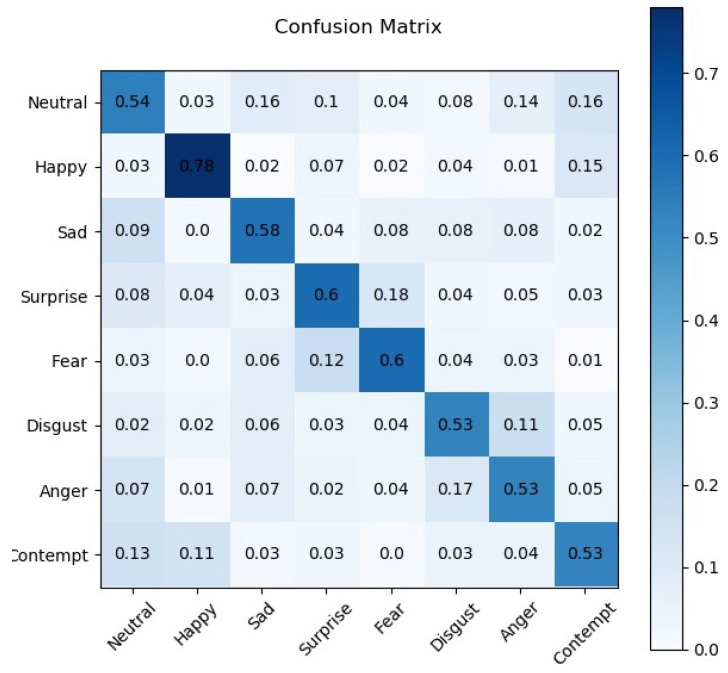
Facial expression classification confusion matrix of the proposed network architecture on AffectNet validation set.

**Facial expression recognition:** To evaluate our proposed light-weight CNN, eight facial expressions in AffectNet validation set are used: Neutral, Happy, Sad, Surprise, Fear, Disgust, Anger and Contempt. Each category of facial expressions contains 500 samples. Figure 4 shows the facial expression classification confusion matrix of the proposed light-weight CNN on AffectNet validation set. Among the eight facial expressions, the highest accuracy is Happy with an accuracy of 78.0%. The accuracies of other facial expressions can also be obtained from the confusion matrix: Neutral (54.4%), Sad (57.8%), Surprise (59.6%), Fear (59.6%), Disgust (53.0%), Anger (53.0%) and Contempt (52.6%). The average accuracy of all eight facial expressions is about 58.50%. We have also evaluated the proposed light-weight CNN by comparing its performance with the state-of-the-art methods including traditional methods and deep learning-based methods. Table 3 shows the results of the comparison on the AffectNet. From Table 3, we can find that our proposed method outperforms other existing methods in terms of classification accuracy on the AffectNet database.

**Valence and arousal estimation:** We have used and calculated 4 different evaluation metrics for evaluation of the valence and arousal estimation task including Root Mean Square Error (RMSE), Pearson’s Correlation Coefficient (CC), Concordance Correlation Coefficient (CCC) and Sign Agreement (SAGR). In order to evaluate the computation complexity, we have also added the number of trainable parameters as the evaluation metric. Table 4 shows the results of our experiments in the valence and arousal estimation on the validation set of the AffectNet databases. We have compared our method with other three state of the art methods which were proposed in [33] and [34]. Mollahosseini et al. [33] utilized both Support Vector Regression (SVR) and AlexNet for valence and arousal estimation. And Siqueira [34] proposed a multi-task learning (MTL) network for this task. From Table 5, it is obvious that the performance of the proposed method outperforms SVR and MTL. Moreover, compared with AlexNet, the proposed method also achieves better performance in arousal estimation on all evaluation metrics. For valence estimation, the performance of AlexNet slightly outperforms our method in valence prediction on all evaluation metrics except for SAGR. But the AlexNet has a high computational cost since the network has about 60 million trainable parameters. In terms of the computation complexity, the proposed light-weight CNN only has about 2 million trainable parameters which are far less than AlexNet.

In summary, the experimental results show that the proposed light-weight CNN can well balance the performance and computation complexity. It achieves superior performance while retaining the low computation complexity compared with state-of-the-art methods. It is thus more suitable for mobile development. Compared with [33], although the proposed approach shows a slightly inferior performance for valence prediction, it has better performance on facial expression recognition and arousal prediction. More importantly, the number of parameters of the proposed method is only about 2 million which is very important for mobile devices, since the storage, memory and computational power of mobile devices are very limited.

## 5. Design for Mobile Platforms

### 5.1. Design Principles and Purpose

To achieve mobile device intelligence using deep learning methods is an emerging trend over recent years. For many mobile applications, the common solutions for achieving mobile device intelligence can be roughly divided into two categories: cloud-based solution and lo-cal-based solution.

The strategy of the local-based solution allows to train a fixed deep learning model such as CNN model offline through a high performance device and then deploy it on mobile devices or the cloud to implement its function. The cloud-based solution allows to upload data to the cloud and then receive results, which may be the best solution for many mobile applications. This is because we don’t need to consider the constrained resources of mobile devices. We can save the storage space and use a high-performance CPU or GPU cluster from the cloud to improve the speed of algorithm execution. However, for facial affective computing, the cloud-based solution is not an ideal solution. There are two weaknesses: (1) privacy issue. For facial affective computing, it is indispensable to capture the facial images of users. Sending users’ facial images to the cloud to analyze facial affect will have potential problems fo privacy security; (2) network latency. Since cloud-based solution needs to upload and receive the data from the cloud, the network environment will be essential for smooth performance. Any data dropout and latency will cause inconvenience when exchanging data.

Therefore, the local-based solution is an alternative solution and more suitable for the facial affective computing task, which could avoid the above issues. The local-based solution which processes the algorithm using local hardware of mobile devices. However, because of the limited storage and computational resources of mobile devices, it is a challenging task to design a CNN architecture and deploy it on mobile devices. In the mobile setting, there is a trade-off between hardware constraints, such as computational resources or storage space, and performance of the CNN models. In this paper, according to the requirements of mobile development, we design a CNN architecture with the goal of balancing this trade-off. We have designed a light-weight CNN architecture for facial affective computing, which maximizes resource utilization and performance on mobile devices. Based on the proposed light-weight CNN, we have developed a facial affective computing application to detect the user’s facial affect in real-time and the application with low hardware requirements can robustly run on the mobile platform. It can benefit many other useful mobile applications such as education, health care, driver monitoring system, and entertainment. In particular, facial affective computing is one of the most important parts of the driver monitoring system, because it can be used to assist safe driving. The drivers require a healthy emotional state during driving for the right judgment of the traffic. The results of facial affective computing can help drivers to recognize their emotions and make drivers aware of them. Therefore, the implemented mobile application for facial affective computing can be applied in the driver monitoring system, which is a good functional component.

### 5.2. Mobile Implementation

We implement a real-time mobile application of facial affective computing on the iOS platform with Swift. The proposed light-weight CNN architecture is deployed on the mobile application by using Apple’s Core ML framework which can integrate trained deep learning models into mobile applications. By using the CPU, GPU, and Neural Engine, Core ML can optimize on-device performance and minimize its memory and power consumption. Core ML is the foundation for mobile development which supports the vision for analyzing images, natural language processing, speech and sound analysis. If the models are created and trained using a supported third-party deep learning framework such as TensorFlow, Core ML framework could provide a conversion tool to convert the trained model to the Core ML model format.

**Figure 5:**
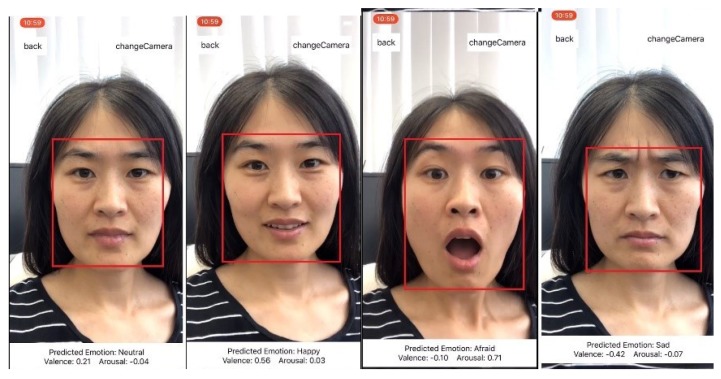
Implementation of the proposed model on the iOS platform.

In our experiment, we first use the human face detection method proposed by Viola and Jones [43] with default parameters for face detection in the mobile application, and then the input facial image is cropped based on the face bounding box and resized to be an equal size of 96 × 96 px. Finally, we analyze the facial affective of the input facial image using the proposed light-weight CNN architecture. We train two separate models for facial expression recognition and valence and arousal estimation tasks. The interface of the implemented mobile application for facial affective computing is shown in Figure 5. The user only needs to grant permission for the application to use the front or back camera of mobile devices. The application could automatically detect the users’ faces and then feed the facial image into the pretrained CNN model to get the results of facial expression, valence and arousal in real-time which are shown on the bottom of the interface.

### 5.3. Evaluation of Storage Consumption and Processing Time

The storage consumption is an important metric for mobile development. For deep learning approaches, we need to train a fixed model and then deploy it on mobile devices to implement its functions. Compared with desktop devices, the storage space of mobile devices is small and limited. However, existing deep learning approaches were mainly designed for desktop devices, which didn’t consider storage consumption of the obtained training model. The size of the pretrained model is so large for mobile devices which can be up to hundreds of megabytes. Moreover, for mobile deployment, different development platforms such as iOS and Android also have different rules about the size of mobile applications. And for many mobile applications, the facial affective computing maybe only one of the functions instead of primary functions, so its storage consumption should be small and reasonable. In order to evaluate the storage consumption of our proposed method, we compare with two classic CNNs in our experiments, AlexNet [44] and VGG16 [37], which are the classic networks for image classification tasks. As shown in Table 6, the model size of them is 233 MB and 528 MB respectively. Compared with two classic CNNs, our proposed light-weight CNN architecture has fewer parameters, and its model size is 15 MB. After transforming to Core ML model format, its size is only 8 MB, approximately two orders of magnitude smaller than two classic CNNs, which meets the requirements of mobile development.

Another problem is to balance performance and computation complexity. For deep learning methods, especially CNNs, the performance of networks depends on the depth of the network structure to some extent. A deeper network structure and higher computation complexity could lead to higher performance. However, high computation complexity means that we need a high-performance device. The mobile devices with hardware constraints cannot guarantee the processing speed while retaining high performance especially for real-time applications. We test our implemented mobile application on two iOS mobile devices, iPhone 5s and iPhone X, which used to represent low-performance and high-performance devices respectively. The results are displayed in Table 5. Although the codes of application are non-optimized, the average running speed on iPhone 5s and iPhone X are 31 fps and 60 fps respectively. For iPhone 5s, the memory consumption is about 100 MB which accounts for 10% of its total memory (1 GB). Since iPhone X has a larger memory than iPhone 5s, the system allocates more memory (150 MB) to the mobile application which ac-counts for 5% of its total memory (3 GB). This means that our proposed light-weight CNN architecture is well suitable for real-time applications on mobile devices.

## 6. Discussion

In this paper, the proposed light-weight CNN architecture achieves high performance when evaluated on the AffectNet database. It can well balance the performance and computational complexity on mobile devices for real-time facial affective computing tasks. The AffectNet database used for training and testing by the proposed method is collected from the wild environment, which provide data variations of many real-life mobile applications. However, we notice that some issues still need to be discussed. First, the proposed method works well when the facial images are visible and frontal, and no occlusion from other objects. However, in some special cases, the method cannot obtain the right results of facial affective. Therefore, designing more effective networks could potentially improve the performance to handle more varied situations from the wild environment. But at the same time, the complexity of the network will also increase. We thus need to find a balance between performance and efficiency on mobile devices. Second, another factor that affects performance is the choice of the face detection method. In our experiment, we used the Viola-Jones face detection algorithm which is a common and popular method but it cannot work well in some cases. One solution is to use the more advanced and effective face detection methods which have been implemented in recent years to lead to good performance. For example, for iOS platforms, Apple provides the Vision framework including a deep learning-based face detection method which can be used in mobile applications in future.

## 7. Conclusions and Future Work

We have proposed a light-weight CNN architecture for real-time facial affective computing on mobile devices. The key design principle of proposed network architecture is to minimize the number of parameters and computational complexity. This network uses facial images as input and outputs the categories of facial expression and values of valance and arousal. Compared to conventional CNNs, the proposed method well balances the high performance and low computation complexity. Moreover, the performance of the proposed method outperforms a series of existing methods in our experiment. We have also implemented a real-time facial affective computing mobile application that has a low consumption of memory and storage on actual mobile devices to demonstrate the feasibility of the proposed method for mobile development.

The performance of our proposed method still has limitations and possibilities for further improvement. In terms of valence prediction, the proposed light-weight CNN architecture still has a gap compared with the method proposed by Mollahosseini et al. [33]. We understand that there is a correlation between facial expression classification and valance/arousal prediction which is the possibility of further improving the performance. The alternative to two separated networks, implementing Multi-Task Learning (MTL) in one network structure is an alternative solution. Future work will address these limitations of the proposed methods and further improve the performance of facial affective computing.

## Figures and Tables

**Table 1:** Details of parameters in proposed network. Notions(channels, kernel, stride) and (kernel, stride) are used to define the conv and pooling layers.

B1	B2	B3
2 × conv layer (32, 3 × 3, 1 × 1) 1 × Maxpooling (2 × 2, 2 × 2)	3 × conv layer (64, 3 × 3, 1 × 1) 1 × Maxpooling (2 × 2, 2 × 2)	3 × conv layer (128, 3 × 3, 1 × 1) 1 × Maxpooling (2 × 2, 2 × 2)
**B4**	**B5**	**B6**
3 × conv layer (256, 3 × 3, 1 × 1)	1 × Global average pooling	Softmax (classification) or linear (regression)

**Table 2:** The numbers of each category of facial expressions in Affectnet for training.

Facial Expression	Number
Neutral	74,874
Happy	134,415
Neutral	25,459
Sad	14,090
Surprise	6,378
Fear	3,803
Disgust	24,882
Contempt	3,750
Total	287,651

**Table 3:** Classification accuracy (%) of facial expressions on Affectnet validation set (the bold value indicates the best classification accuracy).

Methods	Accuracy
Support Vector Machine (Mollahosseini et al. [33])	30.00%
Microsoft Cognitive Services (Mollahosseini et al. [33])	37.00%
AlexNet (Mollahosseini et al. [33])	58.00%
Siqueira [34]	50.32%
Sharma et al. [30]	56.38%
Zeng et al. [31]	57.31%
Ours	**58.50%**

**Table 4:** Comparison of our method with other methods in AffectNet validation set for valence and arousal estimation (the bold value indicates the best result).

Methods	RMSE		CC		SAGR		CCC		Params
	Valence	Arousal	Valence	Arousal	Valence	Arousal	Valence	Arousal	
SVR [33]	0.55	0.42	0.35	0.31	0.57	0.68	0.30	0.18	-
MTL [34]	0.46	**0.37**	-	-	-	-	-	-	50M
AlexNet [33]	**0.37**	0.41	**0.66**	0.54	0.74	0.65	**0.60**	0.34	60M
Ours	0.39	**0.37**	0.61	**0.55**	**0.76**	**0.76**	0.59	**0.48**	**2M**

**Table 5:** Running speed and and memory consumption of our implemented mobile application

Device	Total Memory	Memory Consumption	Running Speed
iPhone 5s	1GB	100 MB	31 fps
iPhone X	3GB	150 MB	60 fps

**Table 6:** Number of parameters and model size of different networks.

Network	Params	Model Size
AlexNet [44]	60 M	233 MB
VGG16 [37]	138 M	528 MB
Light-weight CNN	2 M	15 MB
Light-weight CNN(Core ML)	2 M	8 MB
